# Influence of the Gut Microbiota Composition on Campylobacter jejuni Colonization in Chickens

**DOI:** 10.1128/IAI.00380-17

**Published:** 2017-10-18

**Authors:** Zifeng Han, Thomas Willer, Li Li, Colin Pielsticker, Ivan Rychlik, Philippe Velge, Bernd Kaspers, Silke Rautenschlein

**Affiliations:** a University of Veterinary Medicine Hannover, Clinic for Poultry, Hannover, Germany; b Veterinary Research Institute, Brno, Czech Republic; c INRA, UMR1282 Infectiologie et Santé Publique, Nouzilly, France; d Department for Veterinary Sciences, Institute for Animal Physiology, Faculty of Veterinary Medicine, Ludwig-Maximilians-Universität München, Munich, Germany; University of California, Davis

**Keywords:** Campylobacter jejuni, gut microbiota, immune response

## Abstract

The Campylobacter jejuni-host interaction may be affected by the host's gut microbiota through competitive exclusion, metabolites, or modification of the immune response. To understand this interaction, C. jejuni colonization and local immune responses were compared in chickens with different gut microbiota compositions. Birds were treated with an antibiotic cocktail (AT) (experiments 1 and 2) or raised under germfree (GF) conditions (experiment 3). At 18 days posthatch (dph), they were orally inoculated either with 10^4^ CFU of C. jejuni or with diluent. Cecal as well as systemic C. jejuni colonization, T- and B-cell numbers in the gut, and gut-associated tissue were compared between the different groups. Significantly higher numbers of CFU of C. jejuni were detected in the cecal contents of AT and GF birds, with higher colonization rates in spleen, liver, and ileum, than in birds with a conventional gut microbiota (*P* < 0.05). Significant upregulation of T and B lymphocyte numbers was detected in cecum, cecal tonsils, and bursa of Fabricius of AT or GF birds after C. jejuni inoculation compared to the respective controls (*P* < 0.05). This difference was less clear in birds with a conventional gut microbiota. Histopathological gut lesions were observed only in C. jejuni-inoculated AT and GF birds but not in microbiota-colonized C. jejuni-inoculated hatchmates. These results demonstrate that the gut microbiota may contribute to the control of C. jejuni colonization and prevent lesion development. Further studies are needed to identify key players of the gut microbiota and the mechanisms behind their protective role.

## INTRODUCTION

Campylobacter jejuni is one of the most frequent causative agents of human foodborne gastroenteritis in the world. Chickens, especially meat-type birds, are regarded as the main reservoir for C. jejuni, which may colonize the chicken intestine asymptomatically without significant macroscopical and microscopic lesions (1–3).

The endogenous intestinal microbiota exhibits a high phylogenetic diversity of distinct bacterial species (4–6). The commensal gut microbiota contributes to numerous physiological processes in the host, such as protection of intestinal epithelial cells, digestion of food components, fat and vitamin synthesis, and stimulation of intestinal angiogenesis (4, 5). An additional critical function of the intestinal microbiota is the effective inhibition of colonization and overgrowth of potentially pathogenic microorganisms via competitive exclusion or stimulation of the development of host immune defenses (6).

The role of the gut microbiota in the control of C. jejuni infection has been shown in mice, where it acts as a physical barrier against C. jejuni colonization (7, 8). Germfree (GF) mice and gnotobiotic mice were more susceptible to C. jejuni colonization than mice with a conventional intestinal microbiota. Consequently, ampicillin treatment led to successful C. jejuni colonization of mice (9). Antibiotic-treated (AT) mice showed, indeed, a significantly lower diversity of operational taxonomic units (OTUs) than nontreated mice. Interestingly, mice colonized by a human microbiota were successfully colonized by C. jejuni, in contrast to mice given a mouse microbiota (7). In addition, in Salmonella infection in chickens, it was demonstrated that significantly higher numbers of CFU of Salmonella enterica serovar Typhimurium were detected in 4-day-old birds, after inoculation with *S*. Typhimurium, that were fed with antimicrobial feed additives than chickens that had received a standard diet (8).

The immune system plays an important role in the mucosal defense against bacterial infections. T-cell-mediated immunity was demonstrated in the control of C. jejuni colonization in mice and humans, but little is known about the role of T cells in the control of C. jejuni infection in chickens (10–12). It has been suggested that C. jejuni infections in avian species are associated with Th1 polarization of the immune response (3, 13). It is not fully clear how the gut microbiota may alter the colonization pattern of C. jejuni and may influence the development of local immune responses to C. jejuni colonization.

The goal of this study was to investigate the influence of the intestinal microbiota on C. jejuni colonization in chickens. Birds with a conventional gut microbiota were compared with AT and GF hatchmates. We also investigated and compared the local and systemic immune reactions in C. jejuni-inoculated and C. jejuni-free chickens. Previous studies had suggested a genotype and feed composition influence on the outcome of Campylobacter colonization and immune responses (14, 15). Broiler-type birds, independent of feed composition, mounted a stronger immune response to Campylobacter inoculation than layer-type birds, in which feed composition had a more significant influence on the outcome of pathogen-host interaction (14). But both genotypes were successfully colonized and did not show significant lesions (14). On the other hand, Humphrey et al. (15) and others have shown that different genotypes of meat-type birds varied in colonization pattern and lesion development in experiments using a different dose and strain of Campylobacter from that used in our studies. For this study, we neglected these possible genotype differences because they were not in the focus of our investigations, although AT broiler-type birds were used for experiments 1 and 2, and due to experimental constraints layer-type birds had to be used for experiment 3. Independent of the different genotypes used, our data clearly demonstrate that a conventional intestinal microbiota influences the outcome of C. jejuni colonization as well as the local and systemic immune responses. The mechanisms behind this influencing effect have to be further elucidated, for which these GF and AT chickens may be used as suitable animal models.

## RESULTS

### Reduction of the gut microbiota.

**FIG 1:**
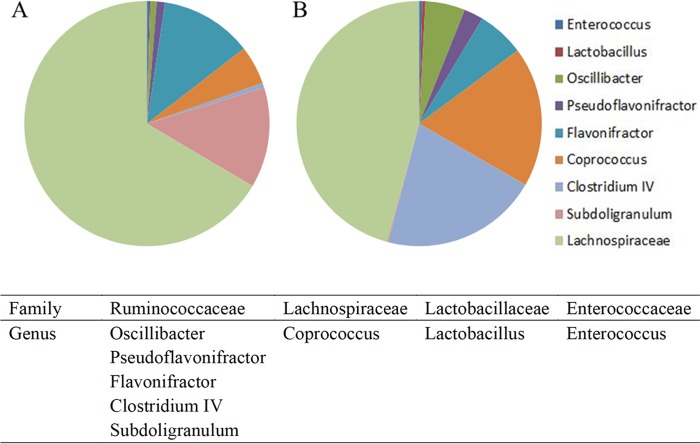
Gut microbiota composition. Taxonomy summary and microbial diversity of operational taxonomic units (OTUs) from cecal samples collected at 25 days posthatch from antibiotic-treated (A) and untreated (B) C. jejuni-free birds in experiment 1 (*n* = 5/group). Charts were generated from raw data, but when we produced them from normalized data, these were essentially the same. We therefore used the maximal data available for each sample.

To confirm the antibiotic effect on the intestinal microbiota, we assessed the diversity of the intestinal bacterial community between AT and untreated broilers at the time of necropsy. At this point, C. jejuni-free control birds had been inoculated with an antibiotic cocktail for 10 days and were exposed to the flora of the housing environment for 14 days (experiment 1) (Fig. 1). Antibiotic treatment of broilers did not lead to a decrease of the overall taxonomical complexity characterized by chao1, equitability, Shannon, or Simpson indices. Despite this, there were 130 OTUs that were differently abundant in control and antibiotic-treated chickens. Moreover, 27 differently abundant OTUs in antibiotic-treated and control chickens ranked among the top 100 most frequent OTUs (data not shown). The sterility of GF chickens in experiment 3 was confirmed weekly by taking fresh fecal droppings, which were incubated under aerobic and anaerobic conditions in tubes containing 10 ml of either sterile brain heart infusion broth or thioglycolate broth with Resazurin. No bacteria, fungi, or yeasts were detected before infection.

### Effect of reduced gut microbiota on C. jejuni colonization.

**FIG 2:**
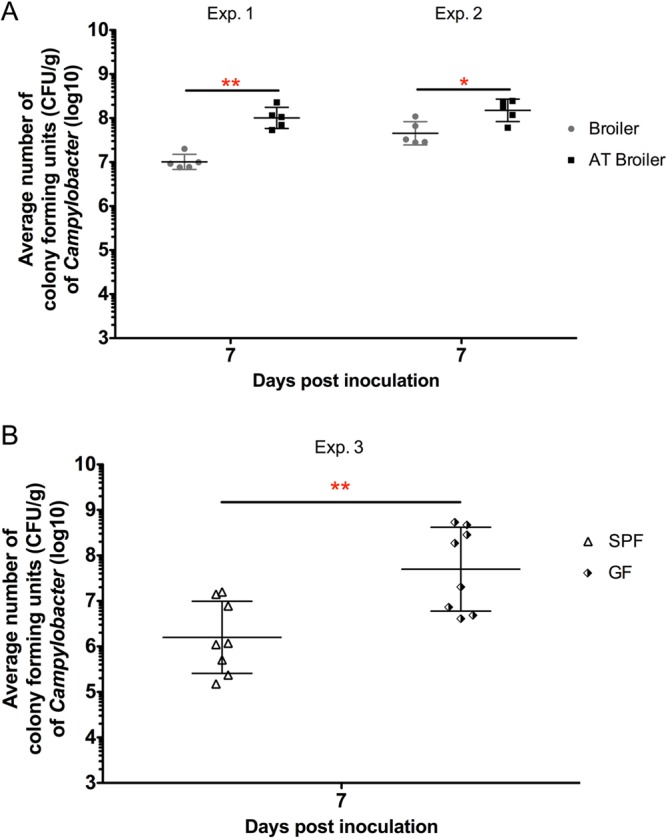
Average CFU of C. jejuni in the cecal content of birds at 7 days after C. jejuni inoculation. (A) CFU of C. jejuni in the cecal content of untreated and antibiotic-treated (AT) C. jejuni-inoculated broilers in experiments 1 and 2 (*n* = 5/group). (B) CFU of C. jejuni in the cecal content of SPF C. jejuni-inoculated birds and germfree (GF) C. jejuni-inoculated birds in experiment 3 (*n* = 8/group). Asterisks indicate significant differences between two C. jejuni-inoculated groups at the indicated days following C. jejuni inoculation: *, *P* < 0.05; **, *P* < 0.01.

Significantly higher numbers of CFU of C. jejuni were observed for C. jejuni-inoculated AT (Fig. 2A) and GF (Fig. 2B) birds than for birds with a “more conventional” gut microbiota. Overall C. jejuni colonization rates of other tissues, including spleen and liver as well as ileum, were higher in AT and GF birds after C. jejuni colonization than in the respective control groups (Table 1).

**TABLE 1:** Qualitative detection of C. jejuni in different tissues^*a*^

Expt no.	Bird groups	No. of C. jejuni-positive tissue samples/total no. tested in:						No. (%) of positive samples/total no. tested
		Spleen	Ileum	Liver	Liver-h*	Heart	Blood	
1	Conv-Lior 6	0/5	4/5	0/5	0/5	0/5	ND	4/25 (16)
	AT-Lior 6	3/5	5/5	3/5	3/5	0/5	ND	14/25 (56)
2	Conv-Lior 6	0/5	5/5	0/5	0/5	0/5	ND	5/25 (20)
	AT-Lior 6	3/5	5/5	2/5	4/5	0/5	ND	14/25 (56)
3	SPF-Lior 6	0/8	3/8	1/8	ND	ND	0/8	4/32 (12.5)
	GF-Lior 6	2/8	8/8	5/8	ND	ND	2/8	17/32 (53.2)

a Abbreviations: Conv, conventional broiler; AT, antibiotic-treated broiler; SPF, specific-pathogen-free birds; GF, germ-free birds; Liver-h*, liver sample was collected and homogenized in 3 ml of PBS; ND, not done.

### C. jejuni elicits a more vigorous immune response in birds that have a reduced or no detectable intestinal microbiota.

**FIG 3:**
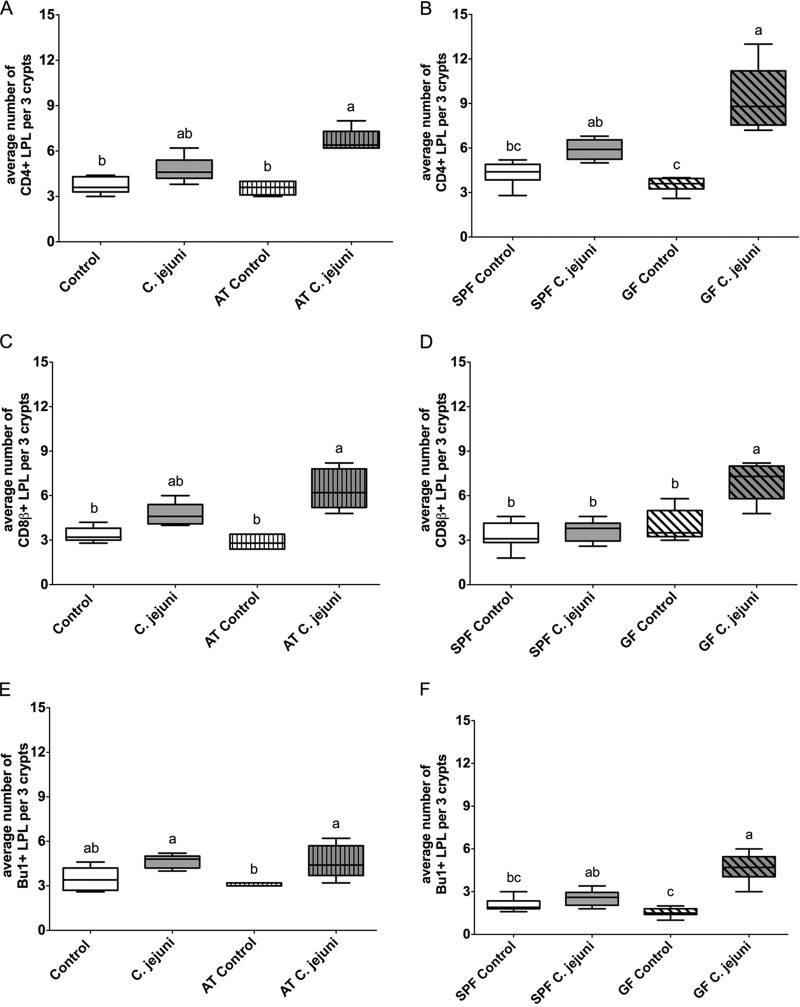
Immunohistochemical detection of T and B lymphocytes in the cecum of birds that were inoculated at 18 dph with either C. jejuni or C. jejuni-free medium. Immunohistochemical detection of CD4^+^ (A and B), CD8β^+^ (C and D), and Bu1^+^ (E and F) lymphocytes in cecal lamina propria. Antibiotic-treated (AT) or untreated commercial broilers in experiment 1 as a representative experiment are presented in panels A, C, and E (*n* = 5/group). Specific-pathogen-free (SPF) and germfree (GF) birds were used in experiment 3 (B, D, and F) (*n* = 8/group). Different lowercase letters (a, b, c) indicate significant differences between groups within the same experiment at the indicated days following C. jejuni inoculation (*P* < 0.05).

**FIG 4:**
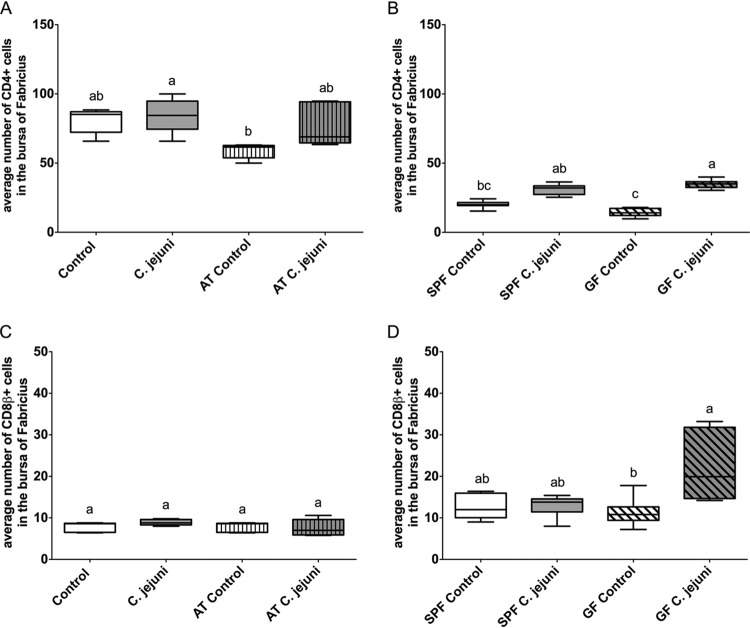
Immunohistochemical detection of T and B lymphocytes in the bursa of Fabricius of birds that had been inoculated with either C. jejuni or C. jejuni-free medium. Immunohistochemical detection of CD4^+^ (A and B) and CD8β^+^ (C and D) T cells in the bursa of Fabricius of birds that had been inoculated with either C. jejuni or C. jejuni-free medium. Antibiotic-treated (AT) or untreated commercial broilers were used in experiments 1 and 2 (experiment 1 used as a representative experiment) (A and C) (*n* = 5/group). SPF and GF birds were used in experiment 3 (B and D) (*n* = 8/group). Different lowercase letters (a, b, c) indicate significant differences between groups at the indicated days following C. jejuni inoculation (*P* < 0.05).

The colonization with a gut microflora slightly influenced the numbers of local T- and B-lymphocyte populations in the cecal lamina propria, cecal tonsil (CT), and bursa of Fabricius. Cell numbers were higher in the cecum, CT, and bursa of Fabricius of birds with a conventional flora than in those of the antibiotic-treated or germfree chickens even in the absence of C. jejuni (Fig. 3 and 4; see also Fig. S2 and S3 in the supplemental material).

C. jejuni-inoculated untreated broilers and specific-pathogen-free (SPF) birds showed, as expected, an increase in the number of T and B lymphocytes in cecum, CT, and bursa of Fabricius compared to the respective control groups, but this increase was not significant (*P* > 0.05) (Fig. 3 and 4; see also Fig. S1 to S3 in the supplemental material).

In all three experiments, significantly higher numbers of cecal CD4^+^, CD8^+^, and B cells were observed in AT and GF birds after C. jejuni inoculation than in AT and GF C. jejuni-free birds, respectively (Fig. 3). This observation for T and B lymphocytes was also found in CT in experiment 3 (Fig. S1 and S2) (CD8^+^ T cell data not shown). Due to the high number and the uneven distribution of immune cells in CT, we did not further quantify the cell populations.

C. jejuni-inoculated AT birds showed in the bursa of Fabricius a clear increase only in the number of CD4^+^ T cells (Fig. 4) and not in the number of CD8^+^ T cells. A significant increase of both the CD4^+^ and CD8^+^ T lymphocytes was observed in the bursa of Fabricius of GF birds after C. jejuni inoculation (Fig. 4 and S3).

### Detection of mRNA expression levels.

**FIG 5:**
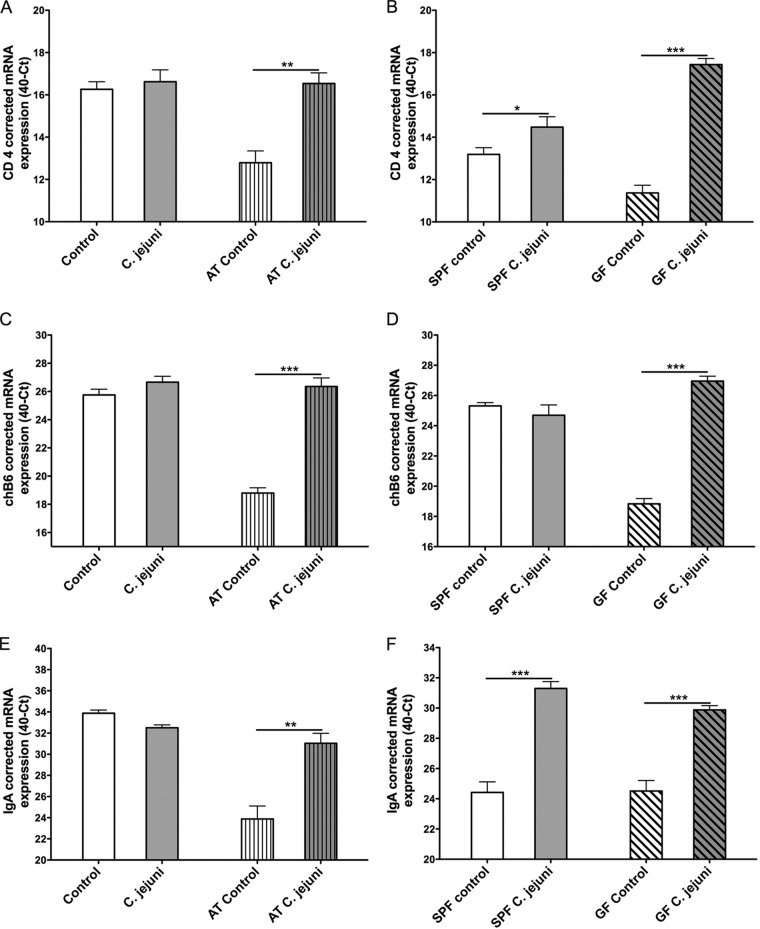
CD4 (A and B), chB6 (C and D), and IgA (E and F) mRNA expression levels in cecum samples of AT or untreated birds (A, C, and D; *n* = 5/group) (experiment 1 used as a representative experiment) and SPF or GF birds (B, D, and F; *n* = 8/group) (experiment 3). Birds were C. jejuni inoculated at 18 dph. Comparison of cytokine mRNA expression levels between C. jejuni-free control and C. jejuni-inoculated birds at 7 dpi. Data are presented as the mean mRNA expression (40-*C_T_*) normalized to 18S. Asterisks indicate significant differences between C. jejuni-inoculated and C. jejuni-free control groups at the indicated days following C. jejuni inoculation: *, *P* < 0.05; **, *P* < 0.01; ***, *P* < 0.001.

To confirm the immunohistochemical data, we determined mRNA expression levels of CD4 and chB6, which relate to T and B cell numbers. To better understand the level of local humoral immunity, we also determined the mRNA expression levels of IgA. C. jejuni-inoculated untreated broilers showed only a slight or no increase in the cecal expression levels of CD4, chB6, and IgA, respectively, compared to noninoculated birds (Fig. 5). C. jejuni-inoculated SPF birds showed significant differences in the cecal expression levels of CD4 and IgA compared to the respective control group (*P* < 0.05) (Fig. 5A and F). Significantly higher levels of CD4 and chB6 as well as IgA mRNA expression were observed in the cecum of C. jejuni-inoculated AT broilers and GF birds than in their C. jejuni-free control groups (*P* < 0.01) (Fig. 5).

### Clinical signs, histological lesions, and goblet cell numbers in the intestines of AT and GF birds after C. jejuni inoculation.

In both experiment 1 and experiment 2, C. jejuni-inoculated AT broilers showed diarrhea, while no clinical signs were observed in C. jejuni-inoculated GF birds in experiment 3.

**FIG 6:**
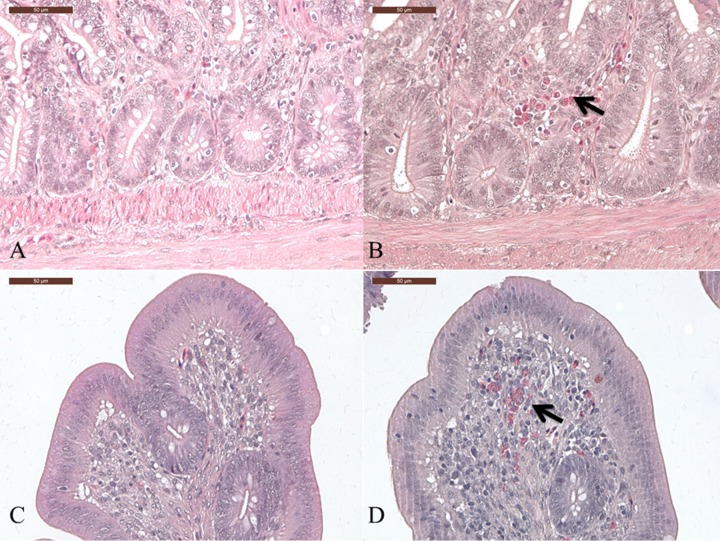
Development of histopathological lesions in the cecum of C. jejuni-free (A and C) and C. jejuni-inoculated (B and D) broilers (A and B) (experiment 1 used as a representative experiment) and SPF birds (C and D) (experiment 3) at 7 days postinoculation. Birds were either AT (experiment 1) or kept under germfree conditions (experiment 3). Shown is the infiltration of heterophils (arrows) in the crypt and villus region of the cecum at 7 days following C. jejuni inoculation.

Microscopically, gut tissue lesions and heterophil infiltration were detected in AT broilers as well as in GF birds after C. jejuni inoculation (Fig. 6). C. jejuni-free birds (Fig. 6A and C) showed low numbers of heterophils (<5/microscopic field; score, 2 [see Materials and Methods for explanation of scores]). We observed moderate and marked (>10/microscopic field; score, 4) infiltration of heterophils in C. jejuni-inoculated AT broilers and GF birds, respectively (data not shown).

**FIG 7:**
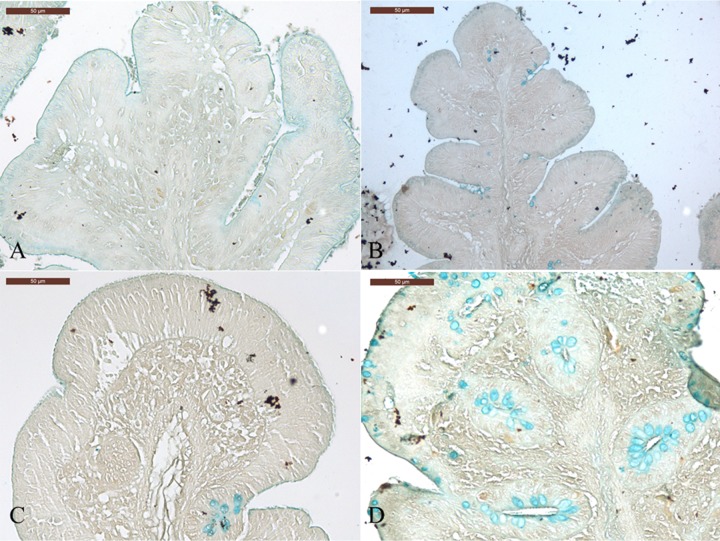
Staining of goblet cells in control (A and C) and C. jejuni-inoculated (B and D) birds at 7 days postinoculation (experiment 3). Cecum section from SPF (A and B) and GF (C and D) birds.

In addition, we also detected higher numbers of goblet cells (score 4) in the cecum of GF birds following C. jejuni inoculation (Fig. 7C and D), but to a lesser extent in SPF birds (score, 2) than in the cecum of C. jejuni-free birds (score, 1) (Fig. 7A and B).

### Effect of antibiotic treatment on gut microbiota composition of commercial broilers after C. jejuni inoculation.

**FIG 8:**
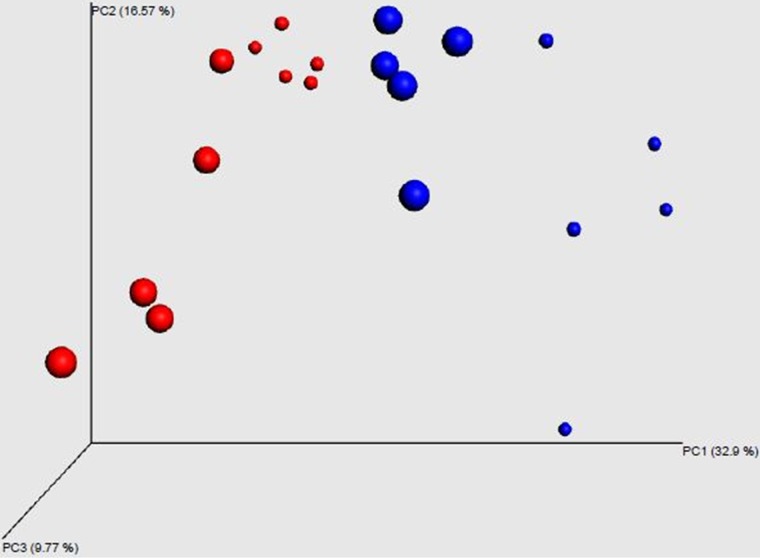
Microbiota diversity. Microbiota diversity in the cecum of antibiotic-treated (red) and untreated (blue) broilers that had been inoculated with either C. jejuni-free medium (small dots) or C. jejuni (big dots) (experiment 1). Images were generated from raw data, but when we produced the images from normalized data, these were essentially the same. We therefore used the maximal data available for each sample.

UniFrac analysis followed by principal-coordinate analysis (PCoA) showed that both antibiotic treatment and C. jejuni colonization affected microbiota composition (Fig. 8). The effect of antibiotic treatment was of a greater consequence than C. jejuni colonization, but there was also a cumulative effect of the treatment and C. jejuni (Fig. 8).

## DISCUSSION

The intestinal microbiota has been considered one of the key factors influencing the host response and outcome of C. jejuni colonization (9, 16, 17). The intestinal microbiota provides opportunistic commensal bacteria that may prevent colonization by enteric bacterial pathogens through activation of the innate and adaptive immune responses as well as antimicrobial defensin production (18). Intestinal microbiota can also contribute to the host defense in multiple ways, such as adjusting the intestinal pH value or changing the limited oxygen level to generate a competitive acidic or unfavorable environment to exogenous pathogens and inhibiting pathogens' attachment to their target sites, as well as limiting fundamentally required nutrients for pathogens (19–21).

It has not been clear to what extent the gut microbiota may affect the C. jejuni colonization in chickens. It was speculated that the gut microbiota may directly or indirectly affect the outcome of C. jejuni colonization through immune modulation. The goal of this study was to compare birds with different gut microbiotas in their C. jejuni colonization pattern and immune responses, which had not been investigated under comparable experimental conditions in chicken before. Three experiments were conducted. In all three experiments, birds were inoculated at the same age (18 days posthatch [dph]) and with the same C. jejuni strain. Due to experimental constraints, some genotype effects cannot be excluded because broiler-type birds were used in experiments 1 and 2, to be able to more closely relate to the field situation, and layer-type birds were used in experiment 3, because this was the available genotype in this germfree system (14, 15), but with respect to the objectives of this study these genotype effects can be neglected.

Consistent with previous investigations (13, 22, 23), none of the C. jejuni-inoculated birds that had a conventional gut microbiota showed clinical signs or pathological or histopathological lesions. Interestingly, diarrhea and marked heterophil infiltration were found in C. jejuni-inoculated AT broilers and GF birds, in contrast to the respective control groups. This is probably related to the fact that intestinal colonization by C. jejuni was higher in AT and GF birds. In poultry, it was demonstrated that the cecal microbiota contributes to the stability of gut health, to host defense, and to pathogen antagonization (24, 25). Following C. jejuni infection, birds may show a higher intestinal permeability and a disrupted intestinal physical barrier allowing translocation of bacteria colonizing the intestine (26). We may speculate that the limited diversity or lack of the cecal microbiota of the AT and GF birds, respectively, may contribute to the higher number of CFU of C. jejuni in the cecum and an enhanced disruption of the intestinal barriers, as indicated by the presence of diarrhea in AT-inoculated birds. The loss of gut integrity and/or the higher bacterial loads may subsequently allow more C. jejuni organisms to cross the intestinal barrier and finally colonize to higher rates other tissues in the AT and GF birds.

This is the first study in chickens with a modified gut flora demonstrating the role of the microbiota on the outcome of C. jejuni colonization. This study reinforces other C. jejuni studies that addressed the role of gut microflora in disease development and pathogenesis in mice (27, 28). Mice with a conventional intestinal microbiota were, indeed, less susceptible to C. jejuni colonization than GF or gnotobiotic ones (7, 9).

The gut microbiota has also been suggested to play a vital role in host immunity (25). In our experiments, we analyzed different immune parameters (CD4, CD8, and B cells) and C. jejuni colonization patterns between birds with conventional and limited gut microbiotas. C. jejuni induced a more vigorous immune response in birds with limited gut microbiota than in conventional birds. In both experiment 1 and experiment 2, C. jejuni-inoculated AT broilers showed higher numbers of T and B lamina propria in the cecum than did untreated birds. The numbers of T lymphocytes in the bursa of Fabricius in C. jejuni-inoculated broilers were also higher than in the C. jejuni-free group. These observations were confirmed in germfree birds (experiment 3), where the differences between C. jejuni-inoculated and noninoculated chickens were even more significant. However, these clear differences were not detected in conventional birds after C. jejuni inoculation. These experiments confirm that independent of the genotype, the gut microbiota may interfere with C. jejuni colonization and also the extent of the subsequent immune response.

To further confirm our immunohistochemical results, we investigated the mRNA expression levels of CD4 and chB6 in the cecum. Consistent with the numbers of cecal lamina propia T and B lymphocytes, significantly lower expression levels of CD4 and chB6 were observed in the cecum of AT and GF birds than in their respective control groups, which were colonized with a microflora after hatch. The expression level of CD4 and chB6 in AT-untreated and SPF birds after C. jejuni inoculation showed no significant or only minor differences compared to the respective control groups. Inoculation of AT and GF birds with C. jejuni led, on the other hand, to a strong increase in gene expression levels compared to the C. jejuni-free controls, supporting the immunohistochemical observation.

C. jejuni can induce circulating IgA in both commercial broilers and SPF birds (29). Significantly higher IgA mRNA expression levels were detected after C. jejuni inoculation of SPF birds as well as AT and GF birds than in the corresponding C. jejuni-free control groups. This observation strengthens the possible controlling role of the gut microbiota on the humoral immune response to C. jejuni colonization. The mechanisms behind this are not clear and have to be elucidated further.

Humphrey and colleagues recently demonstrated that C. jejuni may induce diarrhea, a prolonged inflammatory response, and induction of lymphocyte activation in a specific broiler line (15). The mechanisms behind these differences are not known. Clinical signs were observed only in C. jejuni-inoculated AT broilers (experiment 1 and experiment 2). We may speculate that GF birds did not show any clinical sign due to the breed effect (14). In addition, in all three experiments, AT and GF birds developed histopathological gut tissue lesions and heterophil infiltrations after C. jejuni inoculation, but the respective control groups with a conventional gut microbiota (AT-untreated broilers and SPF birds) did not. Our observations may support others suggesting that C. jejuni is not a commensal but a bacterial pathogen in the chicken gut, but the gut microbiota may prevent lesion development in conventional healthy birds in the field (15, 30). Under field conditions, this delicate balance between gut microbiota, host, and pathogens may easily be disturbed, though, and subsequently subclinical disease may occur and even clinical signs may develop.

It has been demonstrated that the mucus and mucins of the goblet cells provide the first defense line of the gastrointestinal tract and interact with the immune system (31). Interestingly, changes in intestinal microbiota can affect the mucin biosynthesis. A reduction of mucin production has been described for germfree birds (32). In addition, purified chicken mucin was reported to attenuate C. jejuni binding and internalization into HCT-8 cells (33). Another study reported that chicken mucin can reduce the invasion of C. jejuni into intestinal epithelial cells. Furthermore, motility is known to be important for C. jejuni colonization, and binding to chicken mucin has an effect on DNA supercoiling, which alters motility levels of C. jejuni (34). In our study, higher numbers of goblet cells were detected in C. jejuni-inoculated AT and GF birds than in the respective C. jejuni-inoculated control birds, while the effect was more significant in GF chickens. C. jejuni colonization did not affect goblet cell numbers in conventional birds. However, C. jejuni induced an increased number of goblet cells in AT and GF birds, which may subsequently produce mucin as a defense. Further studies should be conducted to investigate mucin composition or production between conventional birds and birds with a limited or absent gut microbiota.

Overall, this study demonstrates that a reduced diversity and population number in the gut microbiota or even the lack of a gut microbiota modulates the pathogenesis of C. jejuni. AT and GF birds are suitable *in vivo* models to identify the role of the intestinal microbiota in C. jejuni colonization in chicken. Our results show a protective role of the gut microbiota on C. jejuni colonization in chickens and contribute to the better understanding of C. jejuni-host interaction. Future studies need to elucidate which bacteria may be the key players in this scenario and further identify possible probiotics candidates to reduce C. jejuni colonization of chickens in the field. Crossover studies using both layer- and meat-type birds in these *in vivo* models may further help to elucidate possible genotype-based differences with respect to microbiota composition, immune responses, and Campylobacter-host interaction.

## MATERIALS AND METHODS

### C. jejuni inoculum preparation.

The C. jejuni strain of serogroup Lior 6 was isolated from a chicken at the Clinic for Poultry, University of Veterinary Medicine Hannover, Hannover, Germany, and was stored in skim milk at −70°C (12).

The cryopreserved bacteria were thawed and plated on charcoal cefoperazone deoxycholate agar (CCDA; Oxoid, Basingstoke, England). The plates were incubated for 48 h under microaerophilic conditions (10% CO_2_, 5% O_2_, 85% hydrogen) at 38°C. After 2 days, one C. jejuni colony was transferred into 3 ml Standard-I-Bouillon (Merck, Darmstadt, Germany) and incubated for another 48 h under microaerophilic conditions at 38°C.

One milliliter of the bacterial suspension was diluted with sterile phosphate-buffered saline (PBS) to achieve approximately 10^4^ CFU/ml for oral inoculation. To confirm the CFU of C. jejuni in the inocula, the bacterial suspension was serially diluted in a 10-fold dilution series, spread on CCDA plates, and incubated for 48 h at 38°C. After incubation, the colonies were counted to calculate the CFU (35).

### Histology.

Samples of the middle region of the cecum, a cecal tonsil, and the bursa of Fabricius were collected, fixed in phosphate-buffered formalin (4%) for 24 h, and further processed for histological examination with standard procedures. The different tissue sections of 2 μm were investigated microscopically for histopathological lesions such as edema in the lamina propria of the cecum, crypt abscesses, and cell ballooning as previously described for mice and chickens after C. jejuni inoculation (22, 36). After the rehydration, sections were stained with 1% alcian blue 8GS (Sigma Co.) for 15 min. Slides were rinsed for 5 min with distilled water, dehydrated with 95% alcohol and 100% alcohol for 2 min each, cleaned with xylene, and then mounted with neutral gums. The goblet cells were counted in five fields per microscopic field (magnification, ×200). Tissue sections were examined in a blinded manner. Lesions were scored by the degree of heterophil infiltration or goblet cell numbers: 1 for a slight increase compared to the respective noninoculated controls, 2 for a mild level of infiltration or low numbers, 3 for moderate levels, 4 for marked levels, and 5 for severe levels of infiltration or high numbers in comparison to the respective noninoculated controls (15).

### Immunohistochemical staining of immune cells.

Frozen sections of middle cecum, cecal tonsil, and bursa of Fabricius were processed as previously described (37, 38). Sections were stained with one of the following mouse anti-chicken unlabeled monoclonal antibodies: anti-CD4, anti-CD8β, and anti-Bu1 (0.05 μg/ml; Southern Biotech, provided by Biozol, Eching, Germany). The secondary anti-mouse IgG biotinylated antibodies and ABC reagent (Vectastain Elite ABC kit; Vector Laboratories Inc., provided by Linaris, Wertheim-Bettingen, Germany) were applied according to the manufacturer's instructions. The enzyme-linked ABC complex was visualized by the reaction with 3.3′-diaminobenzidine (DAB) chromogen substrate and hydrogen peroxide (DAB peroxidase substrate kit; Vector Laboratories Inc.). Sections were examined by light microscopy. The different lymphocyte populations were evaluated by counting the number of stained cells per 3 crypts in the cecal lamina propria and in 5 representative microscopic fields at an optical magnification of ×200 in the bursa of Fabricius of each animal (38).

### qRT-PCR.

For real-time quantitative RT-PCR (qRT-PCR), total RNA was isolated from the middle region of the cecum samples with 1,000 μl Trifast-GOLD reagent (PeqLab, Biotechnologie GmbH, Erlangen, Germany) according to the manufacturer's instructions. RNA quality and concentrations were determined using the NanoDrop ND-1000 system (PeqLab, Biotechnologie GmbH).

All details of the specific primers for the detection of expressed chB6 and IgA as well as the housekeeping gene 18S are described in Table 2. The specific primers for CD4 were obtained from Qiagen Quantitect primer assays. qRT-PCR was performed using the 7300 real-time PCR system (Applied Biosystems, Warrington, UK) with SYBR green as a double-stranded-DNA-specific fluorescent dye. The following cycle profile was applied: 1 cycle at 95°C for 2 min and 40 cycles at 95°C for 15 s, 59°C for 30 s, and 72°C for 30 s, followed by 1 cycle at 95°C for 15 s, 57°C for 30 s, and 95°C for 15 s.

**TABLE 2:** Real-time quantitative RT-PCR primers and probes

Target	Sense^*a*^	Probe/primer sequence (5′–3′)
18S	F	CATGTCTAAGTACACACGGGCGGTA
	R	GGCGCTCGTCGGCATGTATTA
chB6	F	GATCGCCTGCCCTCCAAT
	R	TGGCTTTCCACGTCAGCTATC
IgA	F	CGCCCCTTCCGTCTACGT
	R	CGAAATCGGTTGGTTTTGTTG

a F, forward primer; R, reverse primer.

The results were normalized to the housekeeping gene 18S (39); its expression was comparable between birds of C. jejuni-inoculated and noninoculated groups. Data are expressed as 40 delta threshold cycle (40-*C_T_*) of mRNA expression in the tissues of C. jejuni-inoculated birds and C. jejuni-free controls.

### DNA purification and sequencing of the V3/V4 variable region of the 16S rRNA genes.

Microbiotas were characterized by next-generation sequencing of the V3/V4 variable region of the 16S rRNA genes. Cecal samples were homogenized using zirconia silica beads (BioSpec Products) in a MagNAlyzer (Roche Diagnostics). Following homogenization, the DNA was extracted using the QIAamp DNA Stool minikit according to the manufacturer's instructions (Qiagen). The DNA concentration and quality were determined spectrophotometrically, and the DNA was stored at −20°C until use. Prior to PCR, DNA samples were diluted to 5 ng/μl and used as a template with the forward primer 5′-TCGTCGGCAGCGTCAGATGTGTATAAGAGACAG-MID-*GT-CCTACGGGNGGCWGCAG*-3′ and reverse primer 5′-GTCTCGTGGGCTCGGAGATGTGTATAAGAGACAG-MID-*GT-GACTACHVGGGTATCTAATCC*-3′. The sequences in italics served as an index and an adapter ligation, while underlined sequences allowed an amplification over the V3/V4 region of the 16S rRNA genes. MIDs represent different sequences of 5, 6, 9, or 12 bp in length designed to differentiate samples. PCR amplification and clean-up were done with the Kapa *Taq* HotStart PCR kit (Kapa Biosystems). In the next step, the DNA concentration was determined fluorometrically and the DNA was diluted to 100 ng/μl. Groups of 14 PCR products with the same MID sequence were indexed with a Nextera XT Index kit according to the manufacturer's instructions (Illumina). Prior to sequencing, the concentration of differently indexed samples was determined using a Kapa Library Quantification Complete kit (Kapa Biosystems). All indexed samples were diluted to 4 ng/μl, and 20% of phiX DNA was added. Sequencing was performed using the MiSeq Reagent kit v3 and the MiSEQ apparatus according to the manufacturer's instructions (Illumina).

### Sequence analysis.

Fasta and qual files generated after Illumina sequencing were uploaded into the Qiime software (40). Reverse reads were shortened to a length of 250 bp, and forward and reverse sequences were joined. Quality trimming criteria were set to a value of 19 and no mismatch in the MID sequences. In the next step, chimeric sequences were predicted by slayer algorithm and excluded from subsequent analysis. The resulting sequences were then classified by RDP Seqmatch with an OTU discrimination level set to 97% followed by UniFrac analysis (41). Principal-coordinate analysis (PCoA) was used for data visualization.

### Animals and experimental design. (i) Animals and housing conditions.

For experiments 1 and 2, embryonated eggs from commercial Ross-308 broiler-type chickens were obtained from the BWE Hatchery Weser-Ems GmbH & Co. KG, Visbek, Rechterfeld, Germany. Eggs were incubated and hatched at the Clinic for Poultry, University of Veterinary Medicine Hannover, Hannover, Germany. Chickens were housed and raised at the Clinic for Poultry, University of Veterinary Medicine Hannover.

The birds were assigned randomly to different groups, which were kept in different isolation units to avoid cross-contamination. All units had comparable management conditions. To eradicate the commensal gut microbiota, birds of the AT groups were treated with a cocktail of ampicillin (550 mg/liter; Bela pharm), doxycycline (100 mg/liter; Albrecht), and enrofloxacin (0.3 ml/liter; Bayer) to the drinking water starting at 1 day of life. Antibiotic treatment was maintained for 10 days. At 10 days posthatch, cloacal swabs were collected from each bird/group and spread on Columbia sheep blood agar and cystine lactose electrolyte-deficient agar to confirm the clearing effect of the antibiotic treatment. All samples from birds of the antibiotic-treated group showed no or hardly any bacterial growth after 48 h of incubation under standard protocols, while those of birds of the untreated group showed vigorous growth of different bacterial species, which were not further differentiated, on either agar. From 11 to 18 days dph, birds received water without antibiotics to allow clearance of the substances from the intestinal tract. Feed and water were autoclaved and provided *ad libitum*, and the birds were observed daily for the presence of clinical signs throughout the experiments.

For experiment 3, all White Leghorn chicks (PA12) originated from the same specific-pathogen-free (SPF) flock reared at the infectiology platform PFIE (INRA Val de Loire). The germfree (GF) chickens were obtained by hatching and rearing chickens under sterile conditions as described in reference 42 with some modifications. The surface of the clean eggs, collected just after laying, was sterilized by immersion in 1.5% Divosan (Diversey) for 5 min and for an additional 3 min just before the eggs were transferred into a sterile HEPA-filtered incubator. After 18 days, the egg surface was sterilized in 1.25% Divosan (Diversey) for 4 min at 37°C. Eggs were then transferred to a sterile isolator for hatching. Birds were offered *ad libitum* an X-ray-irradiated starter diet from Special Diets Services (Dietex, Argenteuil, France) and sterilized water for the entire duration of the experiment.

The germfree status of chicks was confirmed regularly by surface swabs of embryonated eggs and after hatch by fecal droppings. Two media were routinely used to determine the lack of bacteria and fungi. Thioglycolate broth with Resazurin (Institute Pasteur Production) was used for sterility tests of samples and for the culture of aerobic, anaerobic, and microaerophilic bacteria. This medium, formulated with pancreatic digest of casein, yeast extract, cystine, and glucose, ensures the growth of a large variety of aerobic and anaerobic bacteria, whereas the addition of sodium thioglycolate at a concentration of 0.05% decreases the redox potential without having a toxic effect. The second medium used (brain heart infusion broth [Difco]) was a highly nutritious medium recommended for the growth of molds and fungi like Aspergillus and fastidious bacteria. Growth of putative microorganisms was monitored for 7 days at 37°C. Birds were additionally tested for the presence of nonculturable bacteria in fecal samples before infection and in cecal samples at the end of the experiment (in noninfected chickens) by quantitative PCR using primers corresponding to “all bacteria” (43). All tested samples were negative for detectable microorganisms by either technique.

### (ii) Experiment 1 design.

Subgroups (*n* = 5) of C. jejuni-free conventional or AT broilers were orally inoculated with C. jejuni strain Lior 6 at a dose of approximately 10^4^ CFU or C. jejuni-free medium at 18 dph by crop inoculation. Five birds of each subgroup were necropsied at 7 days postinoculation (dpi). Histopathological lesions were determined. Heart, spleen, liver, and ileum were investigated for C. jejuni by direct swabs. To avoid cross-contamination, liver samples were collected under sterile conditions immediately at the beginning of the necropsy and subsequently were swabbed from the depth of the parenchyma. In addition, approximately 1-cm^3^ liver sample was collected, homogenized with 3 ml of PBS, and subsequently spread on CCDA plates. The cecal content was analyzed for the number of CFU of C. jejuni/g. Samples of the middle region of the cecum, cecal tonsil (CT), and bursa of Fabricius were taken for immunohistochemical staining of lymphocyte populations. The middle region of the cecum was also evaluated for the expression levels of different immune parameters by using qRT-PCR. In addition, cecal content was collected and investigated for gut microbiota compositions by Illumina sequencing.

### (iii) Experiment 2 design.

Experiment 2 was a repeat of experiment 1. Subgroups (*n* = 5) of birds were orally inoculated with either C. jejuni-free medium or approximately 10^4^ CFU of C. jejuni. Parameters were investigated as described for experiment 1, with the exception that the gut microbiota composition and qRT-PCR analyses were not conducted due to logistical constraints.

### (iv) Experiment 3 design.

Subgroups (*n* = 8) of C. jejuni-free SPF or germfree birds were orally inoculated with C. jejuni-free medium or C. jejuni strain Lior 6 at 18 dph by crop inoculation at a dose of approximately 10^4^ CFU. All birds of each subgroup were necropsied at 7 dpi. The numbers of histopathological lesions and goblet cells were determined. Blood, spleen, liver, and ileum were investigated for C. jejuni by direct swabs. Cecal content was analyzed for the number of CFU of C. jejuni/g. Samples of the middle region of the cecum, cecal tonsil, and bursa of Fabricius were taken for immunohistochemical staining of lymphocyte populations. In addition, middle cecum from birds was evaluated for expression levels of different immune parameters by qRT-PCR as described for experiment 1.

All animal experiments were conducted in accordance with the Animal Welfare Regulations of Lower Saxony and were approved by the Lower Saxony State Office for Customer Protection and Food Safety (LAVES; 33.12 42502-04-13/1215). Animal experiments performed with germfree chicks were carried out at the PFIE platform in strict accordance with French legislation. The specific protocol for the study on germfree chickens was approved by the Ministère de l'Éducation nationale, de l'Enseignement supérieur et de la Recherche (APAFIS#5833-20l60624l6362298). All birds were tested and shown to be negative for C. jejuni by cloacal swabs at the day of C. jejuni inoculation. The animals did not receive any vaccination.

### Statistical analysis.

Statistical analyses were conducted with Statistic version 9.0 (Analytical software, Tallahassee, FL, USA). The Mann-Whitney U test was used for statistical analysis of differences in the CFU numbers of C. jejuni of different C. jejuni-inoculated subgroups at 7 dpi. The differences in the numbers of immune cell populations between C. jejuni-inoculated and noninoculated controls were determined by the Kruskal-Wallis all-pairwise comparison test. The difference in mRNA expression level of immune parameters between C. jejuni-inoculated and C. jejuni-free control groups was determined by the two-sample *t* test or the Wilcoxon rank sum *t* test. Statistical significance was designated as follows: *, *P* < 0.05; **, *P* < 0.01; ***, *P* < 0.001.

## Supplementary Material

Supplemental material

## References

[1] Frost JA 2001 Current epidemiological issues in human campylobacteriosis. Symp Ser Soc Appl Microbiol 2001(30):85S–95S. 10.1046/j.1365-2672.2001.01357.x 11422564

[2] Bronzwaer S, Hugas M, Collins JD, Newell DG, Robinson T, Makela P, Havelaar A 2009 EFSA's 12th Scientific Colloquium—assessing health benefits of controlling Campylobacter in the food chain. Int J Food Microbiol 131:284–285. doi:10.1016/j.ijfoodmicro.2009.01.033. 19246115

[3] Hermans D, Pasmans F, Heyndrickx M, Van Immerseel F, Martel A, Van Deun K, Haesebrouck F 2012 A tolerogenic mucosal immune response leads to persistent Campylobacter jejuni colonization in the chicken gut. Crit Rev Microbiol 38:17–29. doi:10.3109/1040841X.2011.615298. 21995731

[4] Blaut M, Clavel T 2007 Metabolic diversity of the intestinal microbiota: implications for health and disease. J Nutr 137:751S–755S. 17311972 10.1093/jn/137.3.751S

[5] Holmes E, Li JV, Athanasiou T, Ashrafian H, Nicholson JK 2011 Understanding the role of gut microbiome–host metabolic signal disruption in health and disease. Trends Microbiol 19:349–359. doi:10.1016/j.tim.2011.05.006. 21684749

[6] Lawley TD, Walker AW 2013 Intestinal colonization resistance. Immunology 138:1–11. doi:10.1111/j.1365-2567.2012.03616.x. 23240815 PMC3533696

[7] Bereswill S, Fischer A, Plickert R, Haag LM, Otto B, Kuhl AA, Dasti JI, Zautner AE, Munoz M, Loddenkemper C, Gross U, Gobel UB, Heimesaat MM 2011 Novel murine infection models provide deep insights into the “menage a trois” of Campylobacter jejuni, microbiota and host innate immunity. PLoS One 6:e20953. doi:10.1371/journal.pone.0020953. 21698299 PMC3115961

[8] Smith HW, Tucker JF 1978 The effect of antimicrobial feed additives on the colonization of the alimentary tract of chickens by Salmonella typhimurium. J Hyg (Lond) 80:217–231. doi:10.1017/S0022172400053560. 344789 PMC2129995

[9] O'Loughlin JL, Samuelson DR, Braundmeier-Fleming AG, White BA, Haldorson GJ, Stone JB, Lessmann JJ, Eucker TP, Konkel ME 2015 The intestinal microbiota influences Campylobacter jejuni colonization and extraintestinal dissemination in mice. Appl Environ Microbiol 81:4642–4650. doi:10.1128/AEM.00281-15. 25934624 PMC4551207

[10] Smith CK, Kaiser P, Rothwell L, Humphrey T, Barrow PA, Jones MA 2005 Campylobacter jejuni-induced cytokine responses in avian cells. Infect Immun 73:2094–2100. doi:10.1128/IAI.73.4.2094-2100.2005. 15784550 PMC1087459

[11] Jennings JL, Sait LC, Perrett CA, Foster C, Williams LK, Humphrey TJ, Cogan TA 2011 Campylobacter jejuni is associated with, but not sufficient to cause vibrionic hepatitis in chickens. Vet Microbiol 149:193–199. doi:10.1016/j.vetmic.2010.11.005. 21112163

[12] Pielsticker C, Glünder G, Rautenschlein S 2016 Colonization pattern of C. jejuni isolates of human and avian origin and differences in the induction of immune responses in chicken. Vet Immunol Immunopathol 169:1–9. doi:10.1016/j.vetimm.2015.11.005. 26827832

[13] Shaughnessy RG, Meade KG, Cahalane S, Allan B, Reiman C, Callanan JJ, O'Farrelly C 2009 Innate immune gene expression differentiates the early avian intestinal response between Salmonella and Campylobacter. Vet Immunol Immunopathol 132:191–198. doi:10.1016/j.vetimm.2009.06.007. 19632728

[14] Han Z, Willer T, Pielsticker C, Gerzova L, Rychlik I, Rautenschlein S 2016 Differences in host breed and diet influence colonization by Campylobacter jejuni and induction of local immune responses in chicken. Gut Pathog 8:56. doi:10.1186/s13099-016-0133-1. 27843492 PMC5105272

[15] Humphrey S, Chaloner G, Kemmett K, Davidson N, Williams N, Kipar A, Humphrey T, Wigley P 2014 Campylobacter jejuni is not merely a commensal in commercial broiler chickens and affects bird welfare. mBio 5:e01364-14. doi:10.1128/mBio.01364-14. 24987092 PMC4161246

[16] Heimesaat MM, Haag LM, Fischer A, Otto B, Kuhl AA, Gobel UB, Bereswill S 2013 Survey of extra-intestinal immune responses in asymptomatic long-term Campylobacter jejuni-infected mice. Eur J Microbiol Immunol (Bp) 3:174–182. doi:10.1556/EuJMI.3.2013.3.4. 24265935 PMC3832099

[17] Bereswill S, Plickert R, Fischer A, Kuhl AA, Loddenkemper C, Batra A, Siegmund B, Gobel UB, Heimesaat MM 2011 What you eat is what you get: novel Campylobacter models in the quadrangle relationship between nutrition, obesity, microbiota and susceptibility to infection. Eur J Microbiol Immunol (Bp) 1:237–248. doi:10.1556/EuJMI.1.2011.3.8. 24516730 PMC3906620

[18] Diehl GE, Longman RS, Zhang J-X, Breart B, Galan C, Cuesta A, Schwab SR, Littman DR 2013 Microbiota restricts trafficking of bacteria to mesenteric lymph nodes by CX3CR1hi cells. Nature 494:116–120. doi:10.1038/nature11809. 23334413 PMC3711636

[19] Kalliomäki MA, Walker WA 2005 Physiologic and pathologic interactions of bacteria with gastrointestinal epithelium. Gastroenterol Clin North Am 34:383–399. doi:10.1016/j.gtc.2005.05.007. 16084303

[20] Gantois I, Ducatelle R, Pasmans F, Haesebrouck F, Hautefort I, Thompson A, Hinton J, Van Immerseel F 2006 Butyrate specifically down-regulates Salmonella pathogenicity island 1 gene expression. Appl Environ Microbiol 72:946–949. doi:10.1128/AEM.72.1.946-949.2006. 16391141 PMC1352287

[21] Marteyn B, Scorza FB, Sansonetti PJ, Tang C 2011 Breathing life into pathogens: the influence of oxygen on bacterial virulence and host responses in the gastrointestinal tract. Cell Microbiol 13:171–176. doi:10.1111/j.1462-5822.2010.01549.x. 21166974

[22] Beery JT, Hugdahl MB, Doyle MP 1988 Colonization of gastrointestinal tracts of chicks by Campylobacter jejuni. Appl Environ Microbiol 54:2365–2370. 3060015 10.1128/aem.54.10.2365-2370.1988 PMC204261

[23] Dhillon AS, Shivaprasad HL, Schaberg D, Wier F, Weber S, Bandli D 2006 Campylobacter jejuni infection in broiler chickens. Avian Dis 50:55–58. doi:10.1637/7411-071405R.1. 16617982

[24] Rinttilä T, Apajalahti J 2013 Intestinal microbiota and metabolites—implications for broiler chicken health and performance. J Appl Poult Res 22:647–658. doi:10.3382/japr.2013-00742.

[25] Kogut MH 2013 The gut microbiota and host innate immunity: regulators of host metabolism and metabolic diseases in poultry? J Appl Poult Res 22:637–646. doi:10.3382/japr.2013-00741.

[26] Awad WA, Molnar A, Aschenbach JR, Ghareeb K, Khayal B, Hess C, Liebhart D, Dublecz K, Hess M 2015 Campylobacter infection in chickens modulates the intestinal epithelial barrier function. Innate Immun 21:151–160. doi:10.1177/1753425914521648. 24553586

[27] O'Loughlin JL, Samuelson DR, Braundmeier-Fleming AG, White BA, Haldorson GJ, Stone JB, Lessmann JJ, Eucker TP, Konkel ME 2015 The intestinal microbiota influences Campylobacter jejuni colonization and extra-intestinal dissemination in mice. Appl Environ Microbiol doi:10.1128/AEM.00281-15. PMC4551207 25934624

[28] Alutis M, Grundmann U, Fischer A, Kühl A, Bereswill S, Heimesaat M 2014 Selective gelatinase inhibition reduces apoptosis and pro-inflammatory immune cell responses in Campylobacter jejuni-infected gnotobiotic IL-10 deficient mice. Eur J Microbiol Immunol 4:213–222. doi:10.1556/EuJMI-D-14-00031. PMC4271818 25544894

[29] Sahin O, Luo N, Huang S, Zhang Q 2003 Effect of Campylobacter-specific maternal antibodies on Campylobacter jejuni colonization in young chickens. Appl Environ Microbiol 69:5372–5379. doi:10.1128/AEM.69.9.5372-5379.2003. 12957925 PMC194908

[30] Awad WA, Smorodchenko A, Hess C, Aschenbach JR, Molnar A, Dublecz K, Khayal B, Pohl EE, Hess M 2015 Increased intracellular calcium level and impaired nutrient absorption are important pathogenicity traits in the chicken intestinal epithelium during Campylobacter jejuni colonization. Appl Microbiol Biotechnol 99:6431–6441. doi:10.1007/s00253-015-6543-z. 25825050

[31] Pelaseyed T, Bergström JH, Gustafsson JK, Ermund A, Birchenough GM, Schütte A, Post S, Svensson F, Rodríguez-Piñeiro AM, Nyström EE 2014 The mucus and mucins of the goblet cells and enterocytes provide the first defense line of the gastrointestinal tract and interact with the immune system. Immunol Rev 260:8–20. doi:10.1111/imr.12182. 24942678 PMC4281373

[32] Cheled-Shoval S, Gamage NW, Amit-Romach E, Forder R, Marshal J, Van Kessel A, Uni Z 2014 Differences in intestinal mucin dynamics between germ-free and conventionally reared chickens after mannan-oligosaccharide supplementation. Poult Sci 93:636–644. doi:10.3382/ps.2013-03362. 24604857

[33] Alemka A, Whelan S, Gough R, Clyne M, Gallagher ME, Carrington SD, Bourke B 2010 Purified chicken intestinal mucin attenuates Campylobacter jejuni pathogenicity in vitro. J Med Microbiol 59:898–903. doi:10.1099/jmm.0.019315-0. 20466838

[34] Shortt C, Scanlan E, Hilliard A, Cotroneo CE, Bourke B, Cróinín TÓ 2016 DNA supercoiling regulates the motility of Campylobacter jejuni and is altered by growth in the presence of chicken mucus. mBio 7:e01227-16. doi:10.1128/mBio.01227-16. 27624126 PMC5021803

[35] Smith CK, Abuoun M, Cawthraw SA, Humphrey TJ, Rothwell L, Kaiser P, Barrow PA, Jones MA 2008 Campylobacter colonization of the chicken induces a proinflammatory response in mucosal tissues. FEMS Immunol Med Microbiol 54:114–121. doi:10.1111/j.1574-695X.2008.00458.x. 18647351

[36] Murphy H, Cogan T, Humphrey T 2011 Direction of neutrophil movements by Campylobacter-infected intestinal epithelium. Microbes Infect 13:42–48. doi:10.1016/j.micinf.2010.09.007. 20934530

[37] Berndt A, Wilhelm A, Jugert C, Pieper J, Sachse K, Methner U 2007 Chicken cecum immune response to Salmonella enterica serovars of different levels of invasiveness. Infect Immun 75:5993–6007. doi:10.1128/IAI.00695-07. 17709416 PMC2168364

[38] Schwarz A, Gauly M, Abel H, Das G, Humburg J, Rohn K, Breves G, Rautenschlein S 2011 Immunopathogenesis of Ascaridia galli infection in layer chicken. Dev Comp Immunol 35:774–784. doi:10.1016/j.dci.2011.02.012. 21382408

[39] Higgs R, Cormican P, Cahalane S, Allan B, Lloyd AT, Meade K, James T, Lynn DJ, Babiuk LA, O'Farrelly C 2006 Induction of a novel chicken Toll-like receptor following Salmonella enterica serovar Typhimurium infection. Infect Immun 74:1692–1698. doi:10.1128/IAI.74.3.1692-1698.2006. 16495540 PMC1418683

[40] Caporaso JG, Kuczynski J, Stombaugh J, Bittinger K, Bushman FD, Costello EK, Fierer N, Pena AG, Goodrich JK, Gordon JI, Huttley GA, Kelley ST, Knights D, Koenig JE, Ley RE, Lozupone CA, McDonald D, Muegge BD, Pirrung M, Reeder J, Sevinsky JR, Turnbaugh PJ, Walters WA, Widmann J, Yatsunenko T, Zaneveld J, Knight R 2010 QIIME allows analysis of high-throughput community sequencing data. Nat Methods 7:335–336. doi:10.1038/nmeth.f.303. 20383131 PMC3156573

[41] Lozupone C, Lladser ME, Knights D, Stombaugh J, Knight R 2011 UniFrac: an effective distance metric for microbial community comparison. ISME J 5:169–172. doi:10.1038/ismej.2010.133. 20827291 PMC3105689

[42] Schellenberg P, Maillard J 1973 Techniques d'élevage de volailles axéniques. Journées de Recherches Avicoles et Cunicoles INRA-ITAVI-WPSA, p 283–285. ITAVI, Paris, France.

[43] Barman M, Unold D, Shifley K, Amir E, Hung K, Bos N, Salzman N 2008 Enteric salmonellosis disrupts the microbial ecology of the murine gastrointestinal tract. Infect Immun 76:907–915. doi:10.1128/IAI.01432-07. 18160481 PMC2258829

