# Supplementary material for: Influence of the Gut Microbiota Composition on Campylobacter jejuni Colonization in Chickens
Source: Infect Immun. 2017 Oct 18;85(11):e00380-17. doi: 10.1128/IAI.00380-17 (PMC5649013; doi:10.1128/IAI.00380-17)
Supplement: Supplemental material [file supp_85_11_e00380-17__index.html]

Supplemental material 

# Influence of the Gut Microbiota Composition on Campylobacter jejuni Colonization in Chickens

## Supplemental material

- Supplemental file 1 -

  Fig. S1. Immunohistochemical detection of CD4+ lymphocytes in the cecal tonsil of specific pathogen-free and germ-free *C. jejuni*-free control or *C. jejuni*-inoculated birds at 7 days postinoculation (Exp. 3). Fig. S2. Immunohistochemical detection of B lymphocytes in the cecal tonsil of specific pathogen-free and germ-free *C. jejuni*-free control or *C. jejuni*-inoculated birds at 7 days postinoculation (Exp. 3). Fig. S3. Immunohistochemical detection of CD4+ lymphocytes in the bursa of Fabricius of specific pathogen-free and germ-free *C. jejuni*-free control or *C. jejuni*-inoculated birds at 7 days postinoculation (Exp. 3).

  PDF, 2.0M
