# Supplementary material for: Influence of the Gut Microbiota Composition on Campylobacter jejuni Colonization in Chickens
Source: Infect Immun. 2017 Oct 18;85(11):e00380-17. doi: 10.1128/IAI.00380-17 (PMC5649013; doi:10.1128/IAI.00380-17)
Supplement: Supplemental material [file IAI.00380-17_zii999092192s1.pdf]

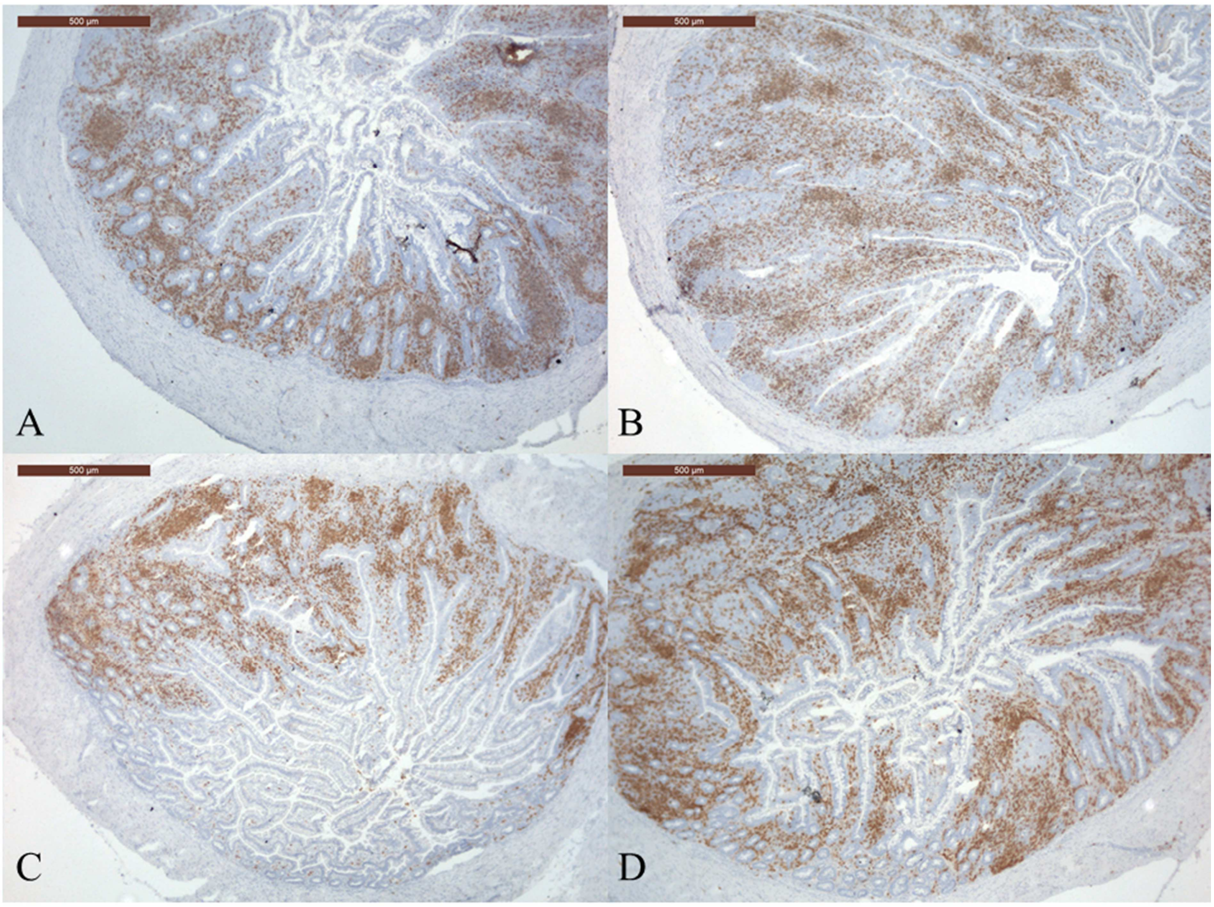

Supplemental FIG 1 Immunohistochemical detection of CD4+ lymphocytes in the caecal tonsil of specific pathogen-free (A and B) and germ-free (C and D) *C. jejuni*-free control (A and C) or *C. jejuni* -inoculated birds (B and D) at 7 days post inoculation (Exp. 3).

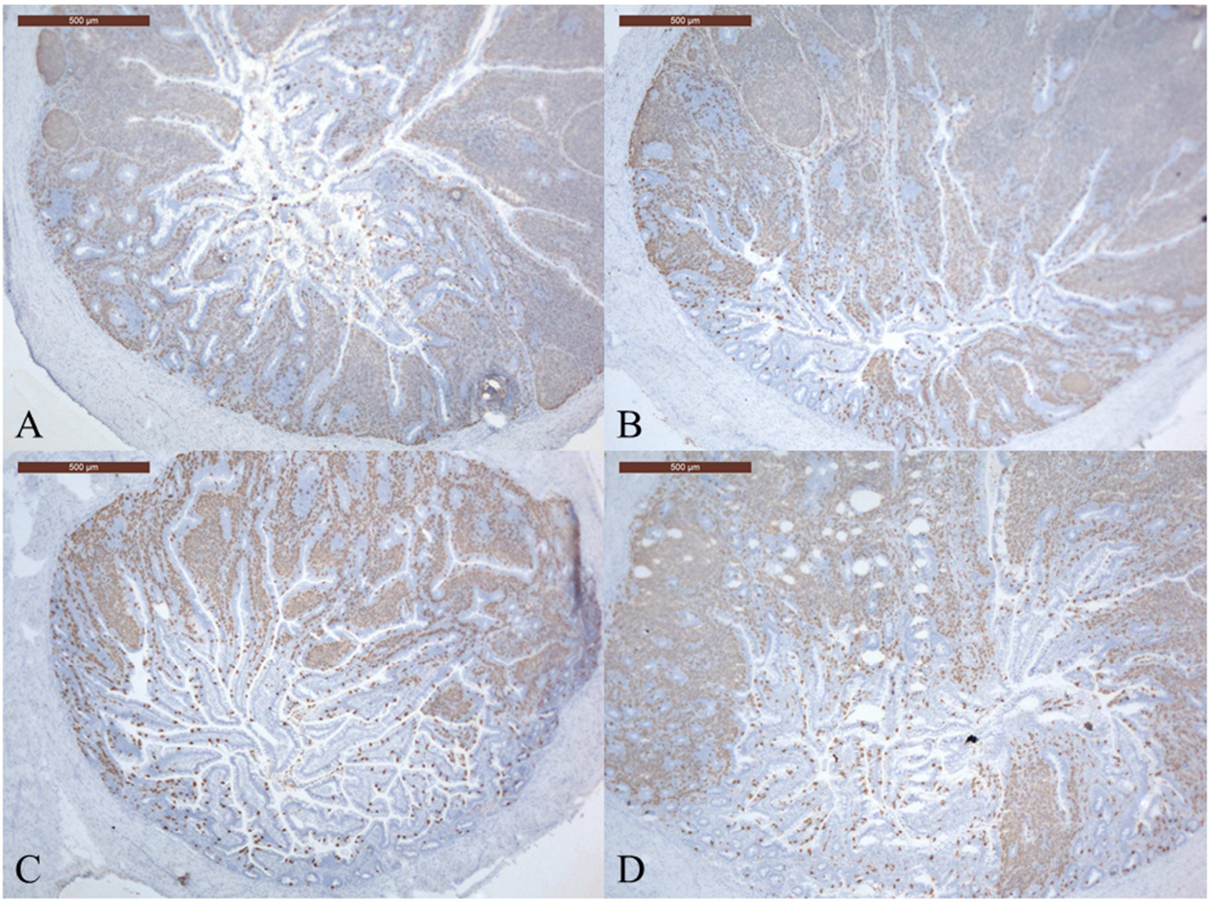

Supplemental FIG 2 Immunohistochemical detection of B lymphocytes in the caecal tonsil of specific pathogen-free (A and B) and germ-free (C and D) *C. jejuni*-free control (A and C) or *C. jejuni* -inoculated birds (B and D) at 7 days post inoculation (Exp. 3).

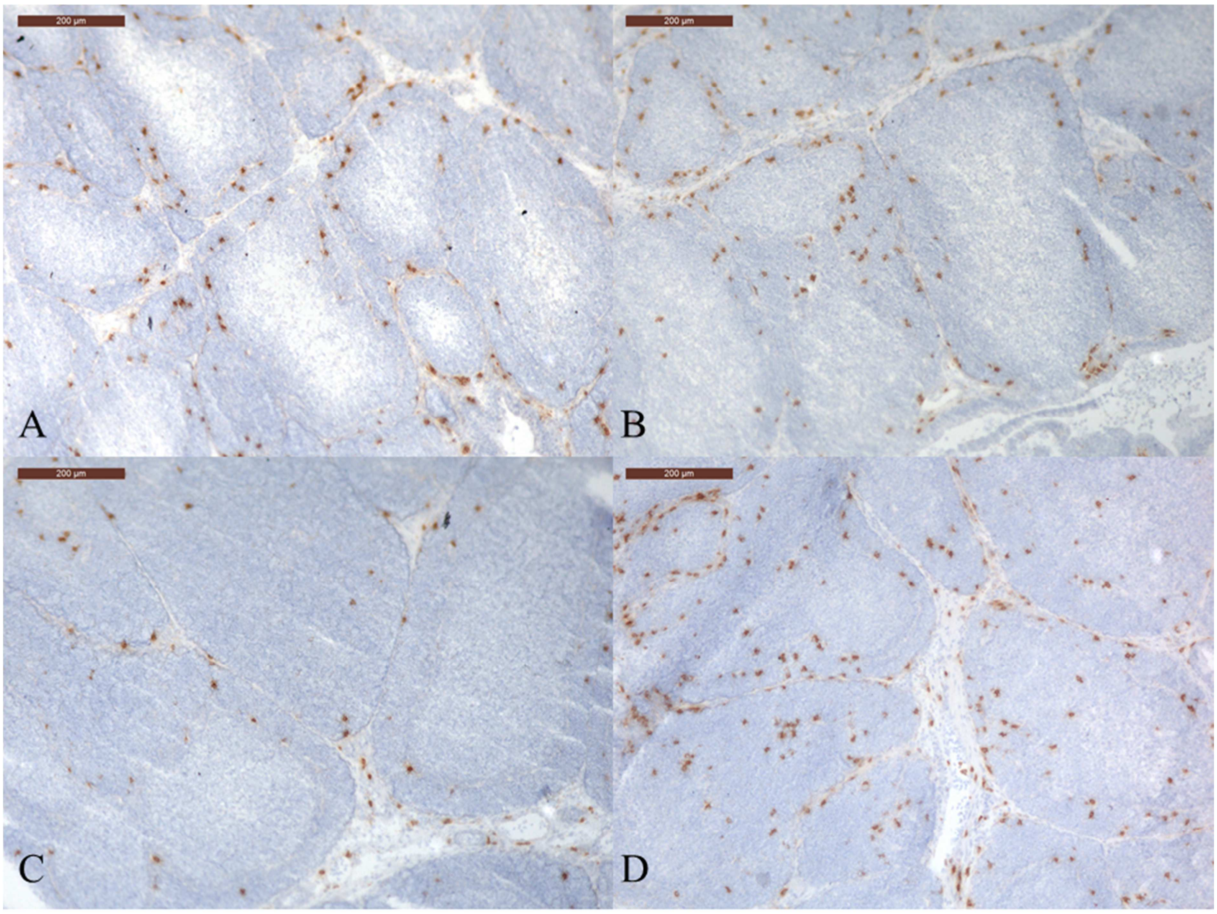

Supplemental FIG 3 Immunohistochemical detection of CD4+ lymphocytes in the bursa of Fabricius of specific pathogen-free (A and B) and germ-free (C and D) *C. jejuni*-free control (A and C) or *C. jejuni* -inoculated birds (B and D) at 7 days post inoculation (Exp. 3).
